# Equity dimensions in initiatives promoting urban health and wellbeing in east and southern Africa

**DOI:** 10.3389/fpubh.2023.1113550

**Published:** 2023-04-11

**Authors:** Rene Loewenson, Gibson Mhlanga, Danny Gotto, Sam Chayikosa, Fastone Goma, Constance Walyaro

**Affiliations:** ^1^Training and Research Support Centre, Harare, Zimbabwe; ^2^Innovations for Development, Kampala, Uganda; ^3^Civic Forum on Human Development, Harare, Zimbabwe; ^4^Centre for Primary Care Research, Lusaka, Zambia; ^5^TalkAB[M]R, Nairobi, Kenya

**Keywords:** urban, equity, health, wellbeing, practice, east and southern Africa

## Abstract

Urbanisation in east and southern Africa (ESA) has brought opportunity and wealth together with multiple dimensions of deprivation. Less well documented in published literature on the ESA region are features of urban practice that promote health equity. This work thus aimed to explore features of urban initiatives aimed at improving health and wellbeing in ESA countries and their contribution to different dimensions of health equity. A thematic analysis was implemented on evidence gathered from 52 documents from online searches and 10 case studies from Harare, Kampala, Lusaka, and Nairobi. Most of the initiatives found focused on social determinants affecting low income communities, particularly water, sanitation, waste management, food security and working and environmental conditions, arising from longstanding urban inequalities and from recent climate and economic challenges. The interventions contributed to changes in social and material conditions and system outcomes. Fewer reported on health status, nutrition, and distributional outcomes. The interventions reported facing contextual, socio-political, institutional, and resource challenges. Various enablers contributed to positive outcomes and helped to address challenges. They included investments in leadership and collective organisation; bringing multiple forms of evidence to planning, including from participatory assessment; building co-design and collaboration across multiple sectors, actors and disciplines; and having credible brokers and processes to catalyse and sustain change. Various forms of mapping and participatory assessment exposed often undocumented shortfalls in conditions affecting health, raising attention to related rights and duties to promote recognitional equity. Investment in social participation, organisation and capacities across the initiatives showed participatory equity to be a consistent feature of promising practice, with both participatory and recognitional equity acting as levers for other dimensions of equity. There was less evidence of distributional, structural and intergenerational equity. However, a focus on low income communities, links made between social, economic and ecological benefit, and investment in women and young people and in urban biodiversity indicated a potential for gains in these areas. The paper discusses learning on local process and design features to strengthen to promote these different dimensions of equity, and issues to address beyond the local level to support such equity-oriented urban initiatives.

## Introduction

Rising urbanization, with urban populations projected to reach 62% of Africa’s population by 2050, are observed by the World Health Organisation (WHO) and UN Habitat to constitute one of the most important global health issues of the 21st century (1). In east and Southern Africa (ESA) countries, urbanisation, while bringing rising and conspicuous wealth for some groups and increasing social connectedness, including through online media, also involves many dimensions of urban stress and deprivation.

Urban areas involve numerous social determinants that can contribute to inequalities in health. Many urban residents in ESA countries live in poor conditions, including substandard and overcrowded housing, water and sanitation systems, unhealthy cooking fuels and technologies, and exposure to health risks from solid waste, air and water pollution, traffic and hazardous working conditions (2–9). Despite being sites of innovation, enterprise and corporate wealth, many urban residents face employment and income insecurity; spend high shares of income on food, utilities and services; and experience rising levels of chronic disease from consumption of poor quality and ultra-processed foods, alcohol, tobacco and other harmful substances (2, 10–12). While services are generally available and geographically accessible, cost, quality and social barriers lead to inverse coverage, especially in meeting the health needs of poorest groups (2, 13–15). Conditions of social insecurity, crime and different forms of violence co-exist with isolation, exclusion and power imbalances within communities and in their interaction with authorities (2).

There is some indication that these determinants and inequalities may be intensifying, making equity a growing issue of concern in urban health (1, 2, 11, 12). Further, these multiple dimensions of social, economic and ecological deficit in urban and peri-urban areas are reported to have been further exacerbated by the COVID-19 pandemic and associated restrictions on movement (16, 17).

While there is documentation of such social determinants of urban inequality, what is less clear, and found to be less well documented in published literature from ESA countries, are features of urbanisation and urban practice that *promote* health equity and wellbeing (2). Various practices are documented to benefit health and wellbeing, with some reports also indicating benefit for low income, marginalised groups. They include urban agriculture (UA) for food security; regulation and taxation of harmful products such as ultra-processed foods, tobacco and alcohol; and health promotion in schools and communities. These approaches have been noted to be more effective when linked with measures that enhance leadership, literacy, social power and autonomy, and when they improve access to appropriate services in disadvantaged groups (2, 12). In the COVID 19 pandemic, initiatives in urban areas that built on prior capacities, processes and relations were able to pivot to a solidarity-driven pandemic response that responded to a range of social needs (18).

The limited documentation of features of equity-promoting practice can be a barrier to their wider application. While acknowledging the general conclusions reached in a global analysis of the drivers of and measures to promote equity in health and wellbeing (19), as a problem statement, the work presented in this paper was motivated by a need to more specifically document and understand the features of initiatives, practices and processes in urban areas of the ESA region that promote health and wellbeing, and from this, to understand those features that address different dimensions of equity. The work was implemented under the umbrella of the Regional Network for Equity in Health in East and Southern Africa (EQUINET) given its long term engagement on health equity, in dialogue with the Accelerating City Equity project of the International Society for Urban Health (ISUH).

Giving a focus to equity, a ‘healthy city’ has been defined as one that enables people to have equitable access to economic opportunities and services; that empowers people to achieve their potential and that nurtures natural environments (20). This resonates with EQUINET’s focus on interventions that seek to allocate resources preferentially to those with the worst health status, backed by a redistribution of social and economic resources and measures to enhance the power and ability people and social groups have to claim rights and make choices over health inputs, and their capacity to use these choices for health and wellbeing (21). While there are various ways of conceptualising the drivers and forms of equity, See and Wilmsen in 2022 suggested a framework that may be used in analysing case studies of different areas of urban health practice (22). Noting a conceptual focus on distributive and procedural justice, they expanded the conceptualisation of equity to include the status, legitimacy and respect different groups have in presenting their interests, and the parity of opportunity they have to be included in decision making. They noted further the need to expose the underlying systemic processes that influence and create an uneven playing field for these other dimensions of equity, as also noted by Anderson as both driver and outcome of social relations that generate prejudice or impose disadvantage (22, 23). Drawing on these conceptualisations (22), and adding the dimension of the longer term impact on future generations and natural resources, we explored five different dimensions of equity, that were also proposed in the ISUH Accelerating City Equity project, and that resonated with the ESA regional understanding of equity. The five dimensions were thus: (i) *participatory or procedural equity*, in terms of groups’ participation in, and their power and influence over decisions; (ii) *recognitional* equity, in terms of formal recognition of the conditions and rights of social groups; (iii) *distributional equity* in terms of the distribution of benefits, burdens and outcomes related to wellbeing; (iv) *structural equity* in terms of underlying policies, laws and norms; and (v) *intergenerational equity* in terms of the benefit for future generations.

## Methods

**Figure 1:**
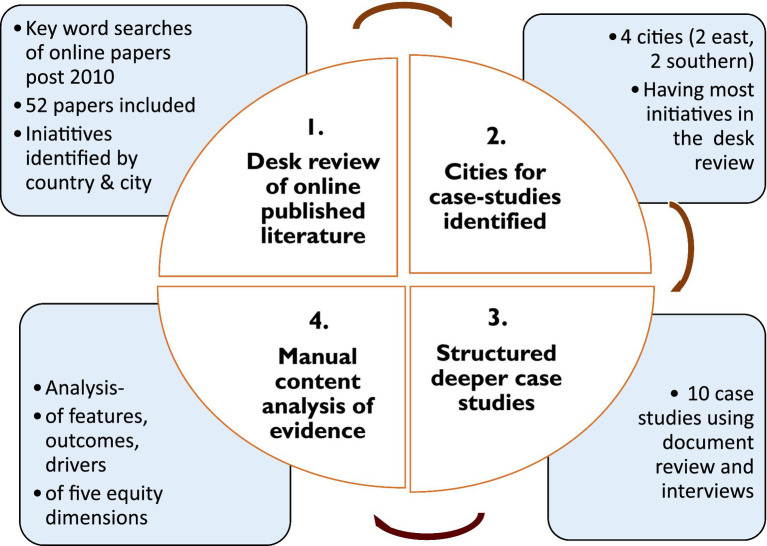
Steps followed in the methods.

Figure 1 summarises the steps followed in the multi-methods approach used.

### Sources of evidence

A document review was implemented in May 2022 to capture evidence on initiatives promoting population health equity in urban/peri-urban areas in the 16 ESA countries covered by EQUINET (Angola, Botswana, DRC, Kenya, Lesotho, Madagascar, Malawi, Mozambique, Mauritius, Namibia, South Africa, Swaziland, Tanzania, Uganda, Zimbabwe, and Zambia). Rather than a systematic review, we used a focused narrative review, followed by structured case studies and content analysis as described below, given the largely qualitative, diverse, and non-biomedical nature of the evidence (24–26). As various methods reviews observe, a content-based focused review may be more usefully applied than a systematic review with such forms of evidence, where the effects are multivariate, the associations non-linear, and where there is need to draw insights both from common findings and from outliers (24–26).

The evidence was drawn from searches of English documents, post 2010, in online journals, Google Scholar, online libraries, and in institutional and international agency websites. For online libraries the search terms were ‘Africa’ or specific country names; ‘urban health’ or ‘wellbeing’ or ‘equity’; and specific social determinants. Of the 196 papers found, 52 were included after review by RL and GM of abstracts and full papers, those in ESA countries relevant to practices promoting urban health and wellbeing included. A manual content analysis was used to extract evidence on features of sustained initiatives aimed at improved urban health and wellbeing, particularly for disadvantaged communities, noting the country and city location. Table 1 presents the distribution of the initiatives documented by country.

**Table 1:** Areas of focus of initiatives found in the desk review (frequencies in brackets).

Country, city	# Papers	Broad area of focus of the initiative
Angola, Luanda (4)	4	Health services, community health workers; citizen generated SDG data for urban planning; low cost housing.
Botswana, Gaborone	1	Decentralised pandemic response, accessible Wi-Fi.
Eswatini, Manzini	1	Use of urban HEART tool to link evidence to planning
Kenya, Nairobi (11)	11	Health services (MCH; health provider training; contraceptive access); Disaggregated evidence for planning; community mapping; food waste management (2); Urban agriculture; right to food; food vendors; household energy; flood management.
Madagascar	1	Mahazoarivo Avarabohitra MCH health service
Malawi Lilongwe (2), Blantyre (1)	3	Health services (contraceptive access, NCD care); Waste management.
Mozambique, Maputo (8), Quelimane (1)	9	Food systems; urban agriculture; waste management; slum upgrading; energy; local architecture; harmful drug use; Citizen data for planning; online communications.
South Africa Ethekwini/Durban (3),Cape Town (1) Johannesburg (2)	6	Health services (family planning, surveillance); Food systems; urban agriculture; Low cost housing; clean environments; transport system.
Tanzania Arusha (1) Kinondoni District (1), not stated (2)	4	Urban PHC; Spatial planning; Greenhouse farming; food safety.
Uganda, Kampala (8), not stated (2)	10	Health needs; refugee wellbeing; community evidence; Food security; urban agriculture; household energy; flooding; waste management.
Zambia Lusaka (4), not stated (2)	6	PHC for NCDs; urban PHC; health system planning; health literacy. Food system; quality sanitation; community electoral voice
Zimbabwe Harare (5), Bulawayo (1) Other (3)	10	Health services (cancer screening; diabetes care; deworming); health committee; gender-sensitive planning. Food waste; solid waste management; community environments; sanitation; clean energy; slum upgrading

Loewenson et al. (27). Some papers included multiple countries (64 examples in 52 papers). No information found for Democratic Republic of Congo, Lesotho, Mauritius, Namibia, and Seychelles.

SDG, Sustainable Development Goals; MCH, Maternal and Child Health; NCD, Non-Communicable Disease; PHC, Primary Health Care.

To explore the findings more deeply, four cities were identified from the document review for detailed case studies in Nairobi, Kenya; Kampala, Uganda; Lusaka, Zambia; and Harare, Zimbabwe. The number of cities was purposively decided on the basis of resource limitations, and to include two from east Africa and two from southern Africa. The selected cities were those that had a higher number of reports in the published literature of sustained initiatives with positive outcomes for disadvantaged communities, where available evidence indicated feasibility for deeper investigation. A local person involved with urban health in each of the four cities (DG, FG, CW, SC), whom we term a ‘focal person’ was involved in the final purposive selection and implementation of the case studies, and all four are co-authors of this paper.

Ten case studies, outlined in Table 2, were produced of specific, sustained urban initiatives across the four cities in June and July 2022, drawing evidence from review of a total of 122 published and grey literature documents relevant to the initiatives. Thirty four (34) interviews were implemented with diverse key informants with direct involvement in the initiatives in the four cities to review, validate and add to findings. The case studies collected evidence on the initiatives in terms of their contextual features, their aims, design and pathways for achieving intended changes, the way equity was addressed; the actors involved and actions implemented. The case studies reported on the time frames, resources and capacities applied, mechanisms and processes, spaces, measures and tools used and the monitoring and review applied, as well as the outcomes achieved. Evidence was included from the documents, key informants and our own analysis of the learning on the drivers, enablers and barriers, equity dimensions addressed and insights that may be transferable to other urban settings. The full versions of the 10 case studies are separately provided (28–31), and a comprehensive report provides detailed information on the methods and empirical findings on the initiatives from both the document review and the case studies (27).

**Table 2:** Case studies by city and areas of work.

Country	# Case studies	Broad area of focus of the initiative
Harare, Zimbabwe	3	Sustainable access to safe clean water and sanitation services Urban agriculture in off-plot farming for income and food security Herbal and nutrition gardening for environmental management
Kampala, Uganda	3	Waste management to address flooding in slum communities Sustainable micro-gardens to address food insecurity Community-led water and sanitation response in informal settlements
Lusaka, Zambia	1	Participatory planning and action by communities and health workers in frontline health services.
Nairobi, Kenya	3	Kibera public space project for multiple services on underused sites Community-led mapping of food vendors in informal settlements Urban agriculture for income, food and ecological security

Chayikosa et al. (28), Goma and Mhlanga (29), Gotto and Mhlanga (30), Walyaro and Mhlanga (31), and Loewenson et al. (27).

As the primary evidence was obtained from public domain secondary evidence from document review, and key informant interaction was implemented after consent for review and validation, this process did not require IRB review. However, we submitted the protocol through ISUH to the New York University IRB for review and received clearance to proceed. The key informant interviews for the case studies used a standard informed consent process, with consent obtained by the focal persons before interviewing key informants, and the key informants anonymised.

## Analysis of the evidence

The evidence for a cross cutting analysis of the (i) common features, (ii) outcomes, (iii) drivers, and (iv) learning noted above was extracted through a manual content analysis within these four key thematic areas from the 10 case studies and 52 papers in the document review, identifying from the evidence further thematic clusters within each major theme.

As noted earlier, a particular focus is given to equity, both in the content analysis and in the discussion of the findings in this paper. The manual thematic content analysis thus identified in the thematic areas above and more directly in the case study texts reference to the five different dimensions of equity explained in the introduction, *viz.*: (i) *participatory or procedural equity* (participation, power and influence in decisions); (ii) *recognitional* equity (formal recognition of the conditions and rights of social groups); (iii) *distributional equity* (in the distribution of benefits, burdens and wellbeing outcomes); (iv) *structural equity* (related to policies, laws and norms); and (v) *intergenerational equity* in terms of the benefit for future generations.

### Limitations

The limited time and resources for the work in 2022 did not allow for deeper searches and snowballing in the desk review, or for wider field interviews, focus group discussions and observations for the case studies. We suggest that further research include these methods. Inclusion of only English language publications in the document review and of case studies from only four cities implies potential linguistic and geographical exclusions that would also need to be addressed in follow up work. Much local work may not be reported in published literature or may exclude negative findings (24–26). However, we based insights on triangulations of different sources, explicitly integrated insights brought by implementers, included negative outcomes in the case studies and subjected the findings to review and validation. The limitations may carry equity implications that should not be lost in the dialogue on and use of the findings. However, we consider the evidence gathered to be sufficient to support the features and insights presented in this paper, particularly those that were most commonly found across multiple case studies or cited sources and settings, while we also note unique experiences and innovations that may demand further research.

## Results

### Focus and outcomes of the initiatives

This section presents the common areas of focus and practices found in the document review, shown earlier in Table 1, and described in the 10 case studies, shown earlier in Table 2, together with the reported outcomes.

The tables indicate a spread of documented work across the region, mainly in the capital cities, usually focused on low income communities and informal settlements, and in some cases, in the peri-urban areas of capital cities. None of the initiatives made specific reference to a theory of change being developed and used to plan and support implementation, although most provided either qualitative outcomes with selected quantitative measures, or tracked targets for outcomes.

#### Areas of focus

As reflected in the two tables, most of the initiatives found take place outside the health system and are *focused on social determinants of health* affecting low income communities, particularly water, sanitation, waste management, energy, land, biodiversity, UA and food safety and security. This common focus suggests these social determinants to be potential priorities in the environments affecting wellbeing in low income communities in the ESA region. Where initiatives involved the health system, this was generally in relation to primary health care or primary care services, and the mechanisms for community engagement with these services.

While social determinants of health dominated, there were other areas of focus. Some initiatives had an explicit *social group focus*, such as work by refugee communities in Uganda to guide newly-arrived urban refugees and support them to overcome language and other barriers in accessing social, financial and other services (32). In this initiative, established refugee communities provide information, guidance and voluntary extension workers to help new arrivals access services in the city. They translate information into accessible languages and help to organise savings groups, given challenges refugees face in opening bank accounts, and advise on local enterprise opportunities (32). A further example of a social group focus was apparent in the community-led participatory mapping of informal food vendors in slum settlements in Nairobi, supported by *Muungano wa Wanavijiji*, the Kenyan federation of slum dwellers (31). This mapping raised the visibility of informal food vendor conditions and their contribution to food security in slums. It improved mutual understanding between vendors, residents and authorities, and brought vendor issues more centrally into the dialogue between communities and government on food systems (31, 33).

Some initiatives had *a system focus*. For example, the Lusaka case study focused on the primary health care (PHC) system in the city, and efforts by the local health authority to integrate community voice in service planning through joint local health worker and community committees; supported by community-driven participatory activities and community photography (photovoice) to bring community voice and priorities to these mechanisms; and health literacy outreach to link committee representatives to informed and active communities (29, 34, 35). Efforts to integrate community voice and build social accountability were found in other initiatives that had a systems focus. Participatory monitoring of waste collection implemented in 42 neighbourhoods of Maputo through a Monitoria Participativa Maputo (MOPA) communications platform sought to improve the city’s waste management systems by enabling greater interaction between marginalised communities and local government (36). In this initiative, once a waste management problem is reported, one of two large waste collection companies and 56 micro-enterprises act to resolve it. The communications platform enables residents to directly notify the municipality of problems, track their resolution and get updates on when, and how, their issue has been addressed (36). In these and other initiatives engaging with systems, the focus was generally on the frontline, primary level services that are closest to communities, using participatory approaches to strengthen social voice and accountability and linking services to social determinants of wellbeing that are prioritised by the low-income communities served (27).

Beyond individual services, a minority of experiences involved integrated area-based approaches. A *spatial focus* in these initiatives enabled a more holistic lens, bringing together multiple services and forms of action, and addressing the multiple needs of low income informal residents. One case study in Nairobi exemplified this well. Kounkuey Design Initiative’s Kibera public space project, initiated in 2016 and ongoing, addresses needs of local residents in Kibera, an informal settlement in Nairobi. It connects residents and local expertise with technical resources to plan with communities and build services and infrastructures in underutlised public sites in Kibera. The activities in the case study integrated river remediation, flood protection; rainwater harvesting; drainage, sanitation and solar energy infrastructures; essential services; WiFi facilities; community buildings and spaces for small businesses and recreation (31, 37, 38). As a spatial initiative, it engaged local residents and diverse actors, resources and services within a defined area to address multiple dimensions of wellbeing, transforming ecosystems, and the built and social environment (31, 37, 38).

While the foci varied, common attention to disadvantaged, marginalised communities in most of the initiatives found implies an engagement with equity, discussed later. The determinants addressed were linked to deeper, often historical or current inequalities in urban development, with poor communities located in low-lying, often informal concentrated settlements affected by flooding and lacking infrastructure, or in areas encroached by land developers (27, 28, 30, 31). Rural–urban migration and rapid urbanisation have increased population density and pressure on infrastructures, exacerbating these conditions. While the experiences point to efforts to mitigate these challenges, discussed later, they also indicated a need for investment in infrastructures, services, land-use planning and legal standards to protect the interests of low income communities. Some of the initiatives strengthened the collective organisation of residents to engage the state on these duties, largely at local level, as a feature of participatory and recognitional equity, further discussed later (27).

#### Areas of change

The processes in the case studies point to the central role of social organisation and participation, both as drivers of change and as outcomes of the initiatives. In cases where interventions were initiated by non-state actors or local councils, social participation by affected groups played a key role in aligning designs to local realities and priorities, in gathering evidence on local conditions and assets, in organising resources such as in savings clubs, and in implementing and reviewing actions (12, 27, 33, 35, 36, 39–41). Deepening cycles of engagement, social confidence and power were noted as outcomes. However, these changes also took time. Many of the initiatives were sustained for more than a decade. Some benefitted from the links between local initiatives and wider social networks such as Slum Dwellers International (SDI) or EQUINET, or with local non-state actors or other cities, to draw on their experience, capacities and tools (27, 29, 31, 33, 39, 42, 43).

The initiatives found reported the achievement of a wide range of other outputs and outcomes. Some interventions achieved outcomes in more than one of the areas noted below, albeit not always formally monitored.

- There were *short and medium-term social changes reported*, such as new skills developed; shifts in attitudes, knowledge and participation by different social groups; a growth in membership of community networks and increased service uptake (18, 29–32, 44).
- There were *material changes visible to communities and authorities*, including a range of improvements in infrastructure, public spaces and services; together with introduction of appropriate technologies and services to address needs; improvements in household incomes; increased organisation of social funds; increased recycling activities and reduced waste dumping (28–31, 37, 43, 45–50).
- There were *longer-term, less easy to measure social, system and material outcomes*, such as increased community self-confidence; strengthened collaboration, solidarity, mutual understanding and improved trust between different social and institutional actors. These outcomes also included increased visibility of conditions affecting low income groups and their inclusion in evidence-based planning and local and wider political and social leadership recognition and support. In addition the findings in some settings showed improved appreciation and marketing of locally-produced fresh foods; reduced food wastage; improved soil quality and biodiversity; and increased pride in neighbourhoods (12, 28–31, 35, 36, 39, 45, 51–53).
- Some case studies identified *health and nutrition outcomes* drawing on routine service data. They reported reduced endemic communicable diseases, nutritional improvements and a decline in seasonal epidemic disease. There were also spill-over effects noted, with uptake of processes and technologies in wider communities, as well as in the social organisation and capacities generated by interventions being used more widely to address other urban challenges (28–30, 35, 54).

### Enablers of and barriers to initiatives for urban health equity

The initiatives found in the document review and case studies gave evidence of a range of enablers and challenges. The more detailed inquiry on these in the 10 case studies is captured in Table 3. This section discusses the more common enablers, and challenges.

**Table 3:** Enablers and barriers in the 10 case study initiatives (key measures shown in bold).

Initiative	Key enablers identified	Barriers/challenges and responses
Harare, Zimbabwe		
Enhancing sustainable access to safe clean water and gender sensitive sanitation services in Epworth	Use of a **local participatory community-based targeting approach to identify beneficiaries** enabled inclusion of vulnerable households, community involvement and ownership. **Working with the community leaders**, CBOs, councilors supported community involvement and sustained actions. **Capacity building of community members** changed social attitudes towards water quality testing and good hygiene practices. **Demonstrating effectiveness** of the intervention facilitated uptake. Central and local government support and participation enabled policy change.	Authorization requirements by authorities at the provincial and district level delayed implementation, but essential as local government participation facilitated policy uptake. Shortfall on resources to meet high demand for the technology. Private sector inputs needed leverage from community or government, municipality, and external funders, with such local resources partially mobilised.
Urban Agriculture In Hatcliffe	Unity, self-determination of community members and a shared purpose to address a key social need. **Formation of Cheziya North Farmers Association** enabled activities, engagement with authorities and leadership, guidance and courage for members to sustain work, despite noted challenges. Political leader perception of the activities as poverty reducing and enabling food security built support. **Free technical support** from the Institute of Engineering staff improved yields. **Fundraising for own projects** and for security for 3 months every year to protect fields.	Contested land, lacking legal title undermined security of tenure. Urban land development reduced land for UA and displaced members. In response, the CNFA organised plot holder agreement to reduce farm sizes and found available adjacent land to accommodate all. Theft of farm produce, overcome by employment of guards for 3 months during the crop season.
Warren Park 2, Herbal and Nutrition Garden	Conducive terrain, climate, soil and water for UA. Availability of land and **lease agreements**. **Willing funding partners** to support the initiative. Willingness of city council to sustain lease of land for UA despite non-payment of costs. Residents’ willingness to offer labour and commitment from the initiators to sustain the initiative during wider socio-political changes. Perceived health benefit of local herbs.	Unaffordable land lease fee led to membership dropout when external funding stopped but **founder commitment** and passion sustained the initiative to bring in new participants. Sustainability affected by weak group cohesion, informal nature and external funder dependency.
Lusaka, Zambia		
Participatory Planning and Action by Communities and Frontline Health Workers in Lusaka	**PRA tools**, community interest and district health management team, ministry of health and Minister support enabled and sustained **repeated and deepening cycles of action and learning** needed for effective community voice and confidence able to influence primary care and community health plans. Election of **community members by the community for committees**, participatory dialogue and input on their **committee constitutions and roles** and **good information flow** between health services and communities built trust. **Mechanisms for exchange across local areas**, like a national meeting of NHCs for sharing of experience and knowledge and to build collective analysis and voice across localities and districts. **Documenting the work**, including online, and involvement in the EQUINET **regional network** widened knowledge, interest, and brought capacities, ideas and respect for the work. The Minister’s pronouncement for nationwide scale-up enabled wide roll-out of the program.	**Legal mandates needed** for NHCs/HCCs post 2006, with guidelines for their functionality. Initial challenges in getting health literacy prioritised at central MoH level, as curative programs often given higher priority. Countered by **ministry champions**, especially by the health minister’s commitment to health literacy. Collaborating partners sometimes had different targets and objectives for participating. A perception of the photovoice as aimed at discrediting the local authority was overcome through community engagement with civic leaders on the issues and the options for and community contributions to resolving them.
Kampala, Uganda		
Sustainable Waste management to address flooding in slum communities of Bwaise III parish	**Co-design with affected communities** meant that members contributions and efforts were valued. Intentional measures for community participation in the design and implementation enhanced buy-in and involvement by the different community members. **Linking waste recycling to a household fuel.** Local village and parish leaders created a supportive environment for implementation. **Private company purchase of products** (briquettes, collected plastics) boosted local income. Absence of affordable energy technologies enabled community adoption of briquettes.	Challenges of space for drying products, connectivity and inadequate services in slums demanded creative measures and continuing engagement with the local authority. Challenges of deficits in slum infrastructures are being addressed through advocacy with the local authority, mayor and councillors on priorities and on benefits for poverty reduction.
Sustainable micro-gardens to address food insecurity in Gayaza parish	**Partnerships with wider stakeholders** including the church, private sector, NGOs and CBOs expanded reach to the most vulnerable, with partners meeting costs of UA inputs and training. Community engagement through the local government, local development agencies and religious institutions, and **collaboration with research institutions enabled access to tested innovations** in UA, enhanced service quality, and boosted production. Access to **a national innovation fund** enabled investment in the initial scale-up phase. Timing during the COVID-19 pandemic meant people were receptive to learning new ways of UA to meet household food needs.	Community discouraged by technology costs and risk of losses due to actions by authorities. Costs reduced by **using local materials**. Absence of water for UA. Addressed through training on **water conservation, harvesting and storage techniques**. Gender norms, weak male involvement, food preferences, household time demands. Rural–urban migration creating land pressure leading to use of wetlands for UA, risking eviction.
Community-led water and sanitation in urban informal settlements	Active and collectively organised engagement and participation of communities was instrumental for resources, self-determined implementation, as was support from and collaboration with local leaders. Infrastructure development **providing local opportunities for jobs, incomes**, and building showed benefit for disadvantaged people.	The COVID-19 pandemic restricted gatherings, halting activities for 4 months. Central level politicians detached from local realities resorting to populism to excite local people used to discredit local initiatives.
Nairobi, Kenya		
Kounkuey Design Initiative’s Kibera public space projects	**Collaborative design** combining capacities, social assets, technical expertise from community leaders, residents and community based organizations. **Productive vibrant and self-sustaining public spaces** as hubs that bring resources and community voice in policy and practice seen to improve livelihoods and service access. Kounkuey **provision of technical skills, negotiating capacities and financial resources** to residents and local CBOs with joint decisions, cultural exchange, and shared responsibility in work. **Kounkuey’s capacities, credibility and reputation** for delivery and management and role in linking residents from informal settlements to official government and agency processes.	Inefficient interventions by local and county governments and limited community access to basic services and infrastructures disrupt social networking and trust, added to by crime and unemployment in the community. Limited data on slum communities weakens community engagement in policy processes. The **consultative and holistic design** of the initiative took these limitations and contexts into account in the design.
Community-led mapping of food vendors in Nairobi’s informal settlements	Commitment to the exercise by Muungano wa Wanavijiji and the residents of the 11 villages The use of **participatory mapping methodologies** and expertise from the institutions vital for effective community and stakeholder engagement and for the success of the study. The use of focus group discussions, and a range of **PRA tools** gave the participants platforms and opportunities to share concerns, experiences and recommendations. Learning **mapping and PRA skills** has helped communities organize collectively and negotiate with other stakeholders, partners and local government for improved services and livelihoods.	Challenges faced by slum dwellers and urban poor people such as exclusion from policy development on key areas where they face deficits or threats, e.g., on slum upgrading, access to services for water, sanitation, transport and energy and electricity and crime and unemployment. The mapping initiative itself generated evidence on these deficits for more formal engagement with duty bearers.
Urban agriculture in Nairobi County	County government interventions supporting resident actions, backed by a **clear legal mandate**, **enabled inter-sectoral capacity building, technical assistance** and **platforms** for further engagement, learning, sharing, action and advocacy on UA, as did partnership with international, national and local organizations, community leaders and CBOs, civil society, academia, and private sector. **Giving focus to equity in decisions** enabled reach to informal settlements.	Poor essential service delivery in informal settlements and cumbersome county operations a barrier to partnerships. Addressed in part by capacity inputs and platform in the design, COVID-19 impacts and climate and weather changes affect gains made in food systems, addressed in part by improving UA practices and food systems.

Chayikosa et al. (28), Goma and Mhlanga (29), Gotto and Mhlanga (30), Walyaro and Mhlanga (31), and Loewenson et al. (27).

CBO, community based organisation; HCC, health centre committee; NHC, neighbourhood health committee; PRA, Participatory reflection and action; WASH, Water, sanitation and hygiene.

#### Enabling leadership and collective organization

The involvement of leaders, members and community-based organisations from affected communities was found to be a common enabler, in processes that listen to community priorities from the onset, and that strengthen collective organisation, capacities and dialogue during implementation. Leadership support from within the community, local authorities and from political and institutional leaders helped to champion initiatives, and sometimes acted as catalysts of or boosts for scale-up of innovation (29, 43, 45, 55) (See Table 3). For example, in Quelimane, Mozambique, the mayor of the city making it his priority to improve public services, infrastructure and food security opened space for various activities to improve solid waste management and UA. Having this leadership support stimulated new forms of collective organisation in urban farmer groups and waste recyclers, and strengthened cooperation between the local government, civil society and the private sector (43).

The commitment of particular individuals within communities played a role in catalysing and sustaining initiatives, especially when challenges arose. For example, in a herbal nutrition garden in Warren Park, a high density housing area of Harare, the persistent support for the initiative by the two founding innovators over more than a decade proved to be important to overcome challenges and to stimulate new ideas to overcome obstacles (28). However the same initiative also indicated that individual leadership maybe insufficient when those involved lack a shared vision and buy-in and are weakly organized collectively, making initiatives vulnerable to disruption (28).

Many of the other experiences thus highlighted the role of investing in social organisation, networking and local capacities for communities to organise evidence, pursue rights claims and engage authorities; as well to enable shared decision-making in response to challenges; and foster solidarity across different social groups in the community (27, 28, 43, 46, 56, 57). In Harare, for example, low-income informal residents in Hatcliffe suburb facing challenges to food and income security organised collectively with support from the Civic Forum on Human Development (CFHD), Zimbabwe Homeless People’s Federation, a community organization, and the International Organization on Migration to identify urban land for collective engagement in off-plot urban agriculture. The residents, coming from a marginalized and disadvantaged community in the city, formed and formally established the ‘Cheziya North Farmers Association’ to build trust and transparency in land allocation to residents for UA. The association provided a sustainable mechanism to support their collective power in facing the significant power imbalances when engaging with local authorities, political actors and land developers. The association was able to leverage collective funding, action and wider institutional contributions for other inputs prioritised by the households involved, including solar powered boreholes, electricity connections and piped water (28).

The initiatives involved processes that explicitly support such collective organisation and agency in communities, including various forms of co-design, literacy, community skills building, and free technical support. The participatory methods and tools used in several initiatives provided collective ways of profiling local lived experience, cultures and knowledge, particularly when embedded in iterative stages of action and learning (58), as exemplified in the strengthened social participation in frontline health services in Lusaka (29, 34). Community confidence was further enabled when processes engaged with communities in their own settings and daily activities, to listen to their views, to use their inputs to adjust design and implementation, and to give feedback on plans (29, 30, 36, 43, 53).

These various participatory and collective processes commonly took place in wider conditions of social and economic insecurity. They are thus not a simple or singular remedy for precariousness. For example, initiatives that relied on community volunteers without making links to improvements in their incomes sometimes overburdened already poor people. This was noted to call for upfront discussion of fairness in the different roles, demands and resources needed for change (59, 60). Local laws and actions by authorities sometimes created obstacles and disempowered local actors and communities (28, 30, 32, 43). While the social organisation discussed earlier helped to address such challenges, social measures do not substitute state duties. As discussed later, deeply rooted problems also call for action from higher level authorities.

#### Bringing multiple forms of evidence to the table

With the gap in disaggregated data in formal systems noted earlier, surveys and other forms of community-led mapping, community surveys, focus group discussions, walk through surveys, photovoice and participatory ways of generating of evidence helped to expose often hidden conditions, strengthening local voice on priorities, and contributing evidence to the co-design, monitoring and review of initiatives (27, 33, 40, 55, 61), as also shown in Table 3. In Matsapha, in Manzini, Eswatini, for example, community and institutional actors in the town used the WHO Urban Health Equity Assessment and Response (Urban HEART) tool to expose gaps in the support provided for wellbeing between urban residents and the large low-income workforce living in the peri-urban fringe areas of the town. The findings on major equity gaps in water, sanitation and waste management; housing, living and neighbourhood conditions; health systems; and access to primary health care were brought to inclusive dialogue to prioritise, plan and implement interventions (55).

Many initiatives integrated ‘listening’ and consultation with affected communities to understand their experience, needs and perceived priorities. This was described, for example, in the consultative processes held by KDI in Kibera (31), or by TAU Uganda and ACTogether in informal settlements in Kampala (30, 62, 63) (See Table 3).

This appeared to be even more powerful when communities were directly involved in gathering, analysing, reporting and using the evidence. In numerous examples, the use of participatory methods enabled lived experience, evidence and analysis to be integrated, using collective processes involving affected communities, and through this building their self-confidence and evidence-base to claim recognition of their priorities (27, 33, 34, 56). Hence, for example, SDI and Muungano wa Wanavijiji, the local informal dweller association in Nairobi, together with informal vendors used their own mapping to profile and negotiate improvements with authorities in their conditions (31, 33), as did community representatives in health centre committees in Lusaka, using participatory research and photovoice (34, 64). These participatory forms of assessment and analysis not only enhanced recognitional equity, but were also reported to strengthen voice in claims over rights and social accountability. This was found, for example in the use by SDI in informal settlements of a ‘right to the city’ lens to map rights violations and duty bearer deficits (65) as well as in forms of appreciative mapping to identify local options and assets that contribute to addressing rights deficits (32, 66). In Maputo’s Monitoria Participativa Maputo (MOPA) example described earlier, the integration of participatory methods within a smartphone based platform helped marginalized communities to link evidence to response from authorities, strengthening social accountability over waste collection in the city (36).

The use of participatory tools in generating evidence to inform interventions signals a respect for community knowledge and experience (58). However, there was less indication of how such evidence interfaced with routine data, or of the weight and value assigned to these different forms of evidence in design of interventions. There was some note of joint monitoring by communities and authorities, such as in assessing water quality in Epworth, bringing shared evidence into planning (28).

#### Co-design and collaboration across multiple actors, sectors, skills and disciplines

The findings indicate that contextual conditions sometimes triggered initiatives, such as in the demand for affordable energy in Kampala, flood management in Nairobi and Kampala, and for UA when the COVID-19 pandemic disrupted food sources in all settings (27).

Initiatives needed to integrate specific measures for communities in precarious situations to have confidence in options. Such measures included initial demonstration by ‘early adopters,’ or bolstering uptake with skills training and resources. In Epworth, a peri-urban, high density low income settlement in Harare metropolitan area, efforts to address deficits in clean water and sanitation in this water-scarce area called for new, water-conserving sanitation technology. A water-conserving toilet innovation was introduced first in 30 pilot households to demonstrate its potential for the wider community. As support for the initiative grew, a ‘lending group’ was formed to fund a locally-driven scale-up (28). Similarly in Gayaza, a peri-urban area of Kampala, the potential for urban micro-gardening was demonstrated by Agriculture for Health and Wealth, a local non-state actor, through a model demonstration site. The site mimicked the small spaces available for low income urban homes, and local leaders and community members were brought to see the options for micro-gardening, with discussion of their situations used to inform outreach (30).

The mix of determinants affecting low income communities called for diverse actions to produce change, even for more focused issues. Measures to produce social improvements were linked in some initiatives to measures for local employment and incomes, supported by affordable technology and training and organisation of new roles; and by various forms of funding to catalyse these opportunities (Table 3) (2, 27, 37, 38, 47). For example in the Gayaza initiative described above, in addition to building capacities for urban micro-gardening in households and schools, the initiative established a shop selling affordable farm inputs. The income from sales and marketing of surplus food from micro-gardens in neighbouring communities helped to resource the activities and improve household income (30). In the Epworth initiative, local youth were trained as pump minders to maintain new water systems, generating income and employment (28). In Bwaise Kampala, an initiative on waste management to address flooding recycled the collected waste to produce briquettes that were then sold locally for household energy use, making links were made between flooding, waste management, waste recycling, energy use, private markets and urban infrastructures (30, 39).

These diverse areas of action called for co-design and collaboration across multiple sectors, actors and disciplines. Area-based approaches, such as in the Kibera public space project described earlier offered space for this, as did having clear and agreed roles and procedures, as described in the joint health service community committees in Lusaka (44) or the management and maintenance of water and sanitation systems in Epworth, Harare (28).

As shown in Table 3, the convening of multi-stakeholder platforms provided a space for dialogue and helped to build shared priorities, as well as support for and the legitimacy of initiatives across the different actors involved, including those contributing technical, technology, financial and enterprise inputs (27, 56). Convening by or with the local authority brought the legal mandate and authority of local government to discussions across sectors, actors and with service providers, as described earlier in Quelimane and Lusaka, or in Nairobi county council’s convening of multiple actors around plans for urban food security (29, 31, 43, 50, 56, 57).

Such multi-actor processes are not always found within local authority cultures. Some initiatives used iterative steps, some moving from informal to formal platforms, demonstrating improvements, to progress what was often gradual institutional change towards a more inclusive governance culture in the city to ‘open up’ to local communities, and to adopt new sources of evidence, measures and approaches (29, 30, 39, 40, 55, 56).

Co-design, co-production and joint review was itself supported by regular information flow between actors, including through representation on committees and dialogue forums, health literacy outreach and exchange visits (29–31). These forms of outreach helped to keep dialogue and planning connected with wider communities. The findings suggest that collaboration was also fostered by joint participation in training activities, by involvement of expertise from different disciplines, and by funding streams that supported cross sectoral innovation (See Table 3) (27, 39, 43).

#### Catalysing and sustaining change

The processes, measures and tools that enable these initiatives often appeared to be catalysed by or to involve values-driven and committed institutions and technical/professional actors. These actors act as credible partners in consulting, information sharing, brokering links and negotiating between communities, authorities, and other agencies (27; Table 3). Hence, for example, the workers within the local health authority in Lusaka played a catalytic and convening role in strengthening community voice in planning and budgeting for frontline health systems in Lusaka (29). The Nairobi county council provided a multi-actor platform to gather and enable dialogue and review across the different organisations and actors leading change around the laws, technologies, systems, production, processing and marketing of interventions for food security in urban Nairobi (31). In some initiatives these catalysts leveraged relevant technologies, resources and inputs to enable options, and demonstrated the feasibility of changes in pilot and demonstration sites (27, 36, 37, 47).

While these key catalysts were generally local, exchange visits across cities and in regional networks gave confidence and ideas to local actors. This was found, for example, in the slum dweller association exchanges between Ghana-Nairobi that triggered the vendor mapping in Nairobi (31); in the EQUINET regional links that brought ideas and experience for the participatory, health literacy and photovoice work in Lusaka (29); or in the co-operation between Milan and Quelimani that shared skills and technologies for urban food security (43).

Various challenges were faced in initiating and sustaining these changes, exemplified in Table 3 for the case studies. Rapid urbanisation, private developments, service declines or rising costs generated pressures and tensions over funds, land and other resources, and with service deficits generated insecurity and frustrations. It was not always easy to leverage private investment and private sector participation (41). Political and social contestation, conflict over land, scarce and unpredictable resource flows, legal and bureaucratic constraints and infrastructure and service deficits presented as challenges in diverse initiatives (27, 67). External project funding often helped to fund innovation, but its unpredictable nature and short term targets were also found to constrain the processes or time needed to build more grounded change (27, 28).

While the enabling features described earlier helped to tackle such challenges, these challenges also demanded strategic and creative responses to sustain initiatives. Being more locally grounded, participatory, and having links to services, systems and local sources of power helped to sustain and even deepen processes, despite challenging conditions and periods. So too did the engagement of higher-level policy actors, or the horizontal spread of practice in iterative steps, providing opportunities to document and profile the changes achieved, drawing in local, national and regional and international support and exchanges (2, 27, 29, 31, 42, 46, 57). The links made between social measures and economic and ecological benefits in disadvantaged communities described earlier also helped to strengthen sustainability. The range of enablers described in the paper appeared to intersect, with multiple levers used, implying that no single enabler can be read as a ‘magic bullet.’ Within contexts of significant and deep inequality, however, the challenges to sustainability cannot be under-estimated.

## Discussion

This section draws on the findings from the document review and case studies to explore their implications for urban health equity. Table 4 summarises the findings in the results within the analytic framework of the five key dimensions of equity.

**Table 4:** Key dimensions of equity in the ESA initiatives.

Initiative	Recognitional equity	Participatory equity	Distributional equity	Structural equity	Intergenerational equity
Harare, Zimbabwe					
Enhancing sustainable access to safe clean water and gender sensitive sanitation services in Epworth	Elevated recognition of deficits in meeting rights to safe water and sanitation. Involved rights to information, participation in planning and management	Established capacities and mechanisms to strengthen inclusion in assessment, planning. Included participatory methods building social power and voice.	Benefit in a low income community with weak links to formal planning systems. Bias towards low income, female and child headed households and people with disabilities.	Linked technology innovation to local health and economic benefit. Policy recognition led to change in WASH approach	Technology innovation conserving water use in area of water stress suggests benefit for future generations.
Urban Agriculture In Hatcliffe	Rights claimed to occupy or lease land, and to UA for household food and incomes.	Establishment of an association to resist powerful political confrontation and address court challenge on land.	Land distribution to low income, food insecure members for UA, but falling land sizes as land taken for urban development.	Land and UA claims raised policy and legal land, food security and welfare system issues.	Assertion of low income land and UA rights relevant to longer term urban development and benefit for future generations.
Warren Park Two, Herbal and Nutrition Garden	Right claimed to land and UA	Shifted from individual towards collective leader-ship, albeit with still weak collec-tive organisation	Benefit to youth, women and elderly in a low-income community	Formal recognition of UA in a five-year renew-able lease, but with high fees.	Youth employment and protection of local indigenous foods sustaining culture.
Lusaka, Zambia					
Participatory planning and action by communities and frontline health workers	Rights recognised to healthy living and social conditions	Right of communities to participate in health service planning and budgeting	Increased focus on social determinants of health prioritised by low income communities	Policy for joint service planning by health workers and community	Reforms to comprehensive PHC that address determinants supports longer term benefit.
Kampala, Uganda					
Sustainable waste management to address flooding in Bwaise III slum communities	Right addressed to healthy, waste and flooding free community environments	Right to design, organise waste management and state duties to provide services. Female leadership	Mapping prioritised the worst affected.	Waste recycling inked to local technology, economy and incomes	Reuse and recycling promoted environmentally sustainable measures.
Sustainable micro-gardens to address food insecurity in Gayaza parish	Right to food and to produce food recognised.	Supported social agency through capacity building, but not in decision-making.	Technology, and support for land constrained low income house-holds	Policy recognition of micro-gardens for UA in high density areas.	Social enterprise as a sustainable model linking social benefit to economic activity.
Community-led water and sanitation response in urban informal settlements	Rights addressed to water and sanitation in slum communities	Organised community-driven structures and measures for information, planning, services for slum-dwellers.	Collectively mobilised local resources to lever wider investments. Equity criteria for inclusion and roles for disadvantaged groups in slums.	A community contracting model used now integrated in government guidelines.	Measures for inclusion of children and youth in technology outreach suggests long term benefits.
Nairobi, Kenya					
Kounkuey Design Initiative’s Kibera public space projects	Rights addressed to healthy public spaces, environments, infrastructure, sanitation, community and small enterprise facilities.	Community networks participated in collaborative project design and planning.	Covered low income informal settlements, especially women and youth. Used evidence, tested ideas to support distributional outcomes. KDI resources matched community labour and in kind inputs.	Led to a new integrated upgrading programme and a Special Planning Area. MoU with Nairobi County to address flood associated risks	Connecting environmental measures to economic opportunities and social capacities, especially in youth, presented a long term model, including to prepare for weather events.
Community-led mapping of food vendors in Nairobi’s informal settlements	Deficits in food security identified, while raising recognition of informal food vendor contributions.	Community-and food vendor led mapping and discussion of findings	The assessment gave voice and evidence to food vendors in engaging on discrimination against them.	Evidence generated used in negotiations by vendors and organisations	Better conditions for informal vendors may be a key determinant of more sustainable urban development.
Urban agriculture in Nairobi County	Rights addressed to food, land and inputs for UA, including as a basis for improved incomes	Local authority led, but involved key Urban stakeholders and community organisations	All residents, especially slum dwellers benefitted, but the distributional impact was unclear.	Strategies identified to implement to UA laws and review land ownership title deeds.	Co-operation between local authorities and stakeholders on sustainable UA and urban ecologies.

Chayikosa et al. (28), Goma and Mhlanga (29), Gotto and Mhlanga (30), Walyaro and Mhlanga (31), and Loewenson et al. (27).

UA, urban agriculture; WASH, Water, sanitation and hygiene; MoU, Memorandum of understanding; KDI, Kounkuey Design Initiative.

An analysis in 2018 of cross-country databases covering ESA countries drawing data from global observatories, UN databases and reported demographic and health surveys found limited disaggregation of evidence within urban areas or by social group (2). Disaggregated evidence that can help to understand distributional outcomes within urban areas is more likely to come from sentinel sites and surveys, and from participatory, qualitative assessments involving those directly affected. However these approaches are more *ad hoc* and limited in coverage, do not use comparable methods or sampling frames across countries, and there was limited evidence found in the 2018 analysis of systematic use of these latter forms of evidence in urban planning (2). This makes it difficult to have a systematic understanding from measured data of equity outcomes within urban areas across ESA countries.

As noted in the methods, the understanding of equity from global analyses, in EQUINET, and in the Accelerating City Equity project covered wider dimensions than the quantitative assessment of distributional health outcomes. The framework applied in analysing the extent to which equity was addressed in the different initiatives, defined and described in the methods, thus included dimensions of recognitional, participatory, distributional, structural and intergenerational equity. The findings discussed in the last section point to areas of progress and deficit in these different dimensions of equity in urban health and wellbeing, as also summarised in Table 4.

### Participatory and recognitional equity as both drivers and outcomes

The common focus in the initiatives on conditions affecting low income and marginalised communities linked recognitional equity to various means of exposing often undocumented shortfalls in living, working and wider conditions affecting health and wellbeing, and using the evidence to raise attention to the implications for rights, duties and areas for change to improve health.

This attention to profiling conditions made various forms of mapping, surveying and assessment a key step across many initiatives, to identify current conditions, to profile prioritised areas of need and those at higher risk, as well as to reveal the social, institutional and other local assets for change. The Manzini, Swaziland use of the urban HEART tool described earlier exemplified this (55). Such assessments have been used to inform the priorities for and design of interventions, to generate socio-political attention to the need for change, and to assess change (27, 40, 61).

Recognitional equity appears to be even more deeply fostered when affected communities themselves are involved in various forms of participatory assessment, as described earlier in the range of examples of work with slum dwellers, informal vendors, low income residents, urban farmers and others. They use participatory surveys and mapping (31, 33), participatory action research and photovoice (34, 64), or smartphone based applications (36). Involving local people in the generation and analysis of evidence not only informed initiatives, but built social confidence for those affected to claim recognition of their priorities, particularly when linked to rights based approaches, as described earlier in claims on rights to food, to the city, or to local authority accountability for services (36, 53, 65). Listening to communities from the onset, consulting those affected during the processes, and more deeply organising evidence and analysis within and from affected groups brought new evidence to planning processes and recognition of the local assets that contribute to addressing them (27, 32). It thus made the design of initiatives more relevant to the local situations, and built ownership of these initiatives amongst those affected (27).

Beyond participation in bringing evidence to stimulate, frame or track initiatives, the findings also point to the critical role of investments in wider forms of social participation in many of the initiatives. As earlier described, the processes establish, and strengthen inclusion in and capacities of mechanisms and dialogue forums. They also provide a range of training and capacity building activities to enable intervention by communities in these forums (27, 53, 57).

Many of the initiatives described in the findings strengthened associational networking, organisation and collective leadership, to engage within the processes, in the mechanisms for dialogue, and to support the collective social power for those in precarious conditions to negotiate claims, face challenges and manage contestation (27), such as detailed earlier in the example of the Cheziya North Farmers Association in Harare (28). Local political and institutional champions helped to support these forms of collective capacity building, organisation and voice in the community, as described in Lusaka and Quelimane (29, 43).

The common investment in various forms of social participation, organisation and capacities across the initiatives highlight performance on participatory equity as a relatively consistent and potentially central feature of promising practice in urban wellbeing. A growth in social organisation, power, self-confidence and engagement with and influence over decisions in the initiatives points to gains in participatory equity. Such gains were not simply outcomes in their own right, but also appeared to be important levers for other dimensions of equity.

### Distributional equity implicit in the focus and design

In a context of limited disaggregated routine data in cities, the various areas of improvement noted in the findings suggest gains in distributional equity. Most initiatives poorly captured the relative gains for different social groups through monitoring or routine data. Some initiatives included research to formally assess differential health outcomes related to specific urban services, albeit without attributing to specific features of interventions (4, 13, 54, 66). Embedding more systematic and distributional performance and outcome monitoring in initiatives for urban wellbeing and improving the within-area disaggregation of routine data systems would appear to be a significant area for further development.

The location of a majority of the initiatives in disadvantaged communities, such as low income and informal residents, precarious workers, and those with least social power, and the explicit intention of interventions to address various drivers of disadvantage indicated an intention to support distributional equity.

Many initiatives described in the findings thus addressed distributional equity by improving wellbeing for specific marginalised communities. For example, in the context of Uganda’s policy of integrating refugees into existing urban communities, the initiative linking newly arrived refuges with existing networks of refugee communities described earlier helped to address the various dimensions of the disadvantage they face (32).

Where initiatives and services covered the entire population of the area and intended to benefit all in the community, specific measures were included to facilitate benefit for specific groups, such as women, youth, elderly people, or people living with disabilities (29, 31, 50, 54). The various examples described earlier, including those detailed in Epworth, Lusaka, Gayaza and Bwaise Kampala (28–30) indicate that this involved outreach to, literacy and training in and involvement of leadership from disadvantaged social groups and areas; demonstration sites and uptake by ‘early adopters’ to boost confidence in options for those in precarious conditions, and bolstering their uptake with skills training and resources. Linking social improvements to measures for local employment and incomes, supported by affordable technology and various forms of funding also helped to catalyse and sustain involvement and uptake by those in insecure economic conditions, such as women farmers in urban slums, and unemployed youth.

Making such links between social, ecological and economic benefits appeared to be important for distributional equity. As noted later, this feature also has pertinence in addressing structural equity. While barriers such as land development, resource deficits, conflict with authorities and legal challenges acted to weaken distributional equity, they were countered by measures that linked social interventions to local organisation, and to opportunities for employment and incomes (2, 27, 39, 45, 49).

### Investing in youth and sustainable models for intergenerational equity

Intergenerational equity was not noted as a specific goal in the initiatives found. It was however integrated through investments that sought to protect urban biodiversity and environments; or to apply sustainable approaches for urban agriculture, waste management and recycling (27, 53, 54). A number of initiatives included investments in youth capacities and roles, such as the role of youth pump-minders in Epworth, Harare. Such investments could enhance intergenerational equity. Some initiatives have more explicitly engaged young people on their futures. In participatory dialogues, different groups of young people in Harare and Lusaka identified their perceived priorities for health today, and those that they saw as becoming more critical in the coming decades, engaging on these with the local authority (2). Also in Lusaka, a ‘food change lab’ that brought youth together with other food system stakeholders in the city, under the banner of ‘Youth for Sustainable Food Zambia’ discussed how to meet current and future needs for healthy food in the city, particularly for low-income consumers (53). These are isolated examples. There appears to be scope for more explicit integration in urban initiatives of applying a ‘future lens’ to assess how far approaches address projected risks in the future, and to integrate the voice and role of young people in this and in decisions that affect them.

### Assets for and challenges in addressing structural equity

The initiatives outlined in the findings raise policy or legal issues relevant to structural equity, There was, however, limited report of policy change. One example of such policy change was in ACTogether’s work with the National Slum Dwellers Federation of Uganda and partners to improve water and sanitation in Kampala slums, where a community contracting model was developed for informal residents to contract builders from amongst marginalised members. This contributed to a new policy framework by Uganda’s Ministry of Lands Housing and Urban Development to guide community contracting (30). While the findings indicate examples of authorities waiving land leases or enabling more inclusive dialogue, there are fewer examples of such formal changes in national procedures.

Local initiatives in precarious communities in ESA face challenges in addressing structural equity, given longstanding insecurity and barriers to self-determined action, organisation and initiative, wider top-down hierarchies of power in planning and regulatory systems and their enforcement, and unpredictable financing and socio-political volatility that disrupts the time needed for improvement cycles and achievements to build deeper changes (27, 28, 48, 50). As Anderson notes, systemic or structural inequities drive or reinforce social relations that exclude and disadvantage some population groups, entrench stigmatising representations of these groups in public discourse and perpetuate their exclusion from state and private forums where decisions are made. Confronting such inequities is thus argued to be critical for democratic society (23).

The findings highlight how some processes bring together several sources of power and resources around shared goals to address these challenges. These processes converge leadership or champions from within communities, civil society, technical agencies and local governments around shared goals and actions, and involve institutions able to engage and promote the benefits and wider application of the work with higher levels of power and authority (27).

Some initiatives showed evidence of new approaches in urban economies and services through the local development and introduction of appropriate and accessible technologies. This was noted for example in actions described in the findings on water and sanitation systems, on waste management, on food systems, communication applications and equipment for health services (27, 45, 48, 63, 68).

Many of these technological and material approaches were self-initiated within communities, as a direct response to local conditions, or developed and introduced by local institutions working with communities. The evidence highlights the potential for community and local innovation. It also suggests that gains in equity in technology innovations maybe more sustained where there is local control over the design and production of technology, to ensure its relevance and accessibility, and to link technologies to local employment and incomes for disadvantaged groups, with support from wider skills processes. With local levels often innovating but having limited authority and scope to address many of these policy-related issues, it is argued that technology as a support for equity cannot be left as a micro-issue, and needs to be linked to wider urban planning systems and services and to national resources for innovation.

### Learning and insights on improving urban health equity

The findings clearly indicate that equity-oriented action and change in urban areas is both necessary and possible. While not always explicitly addressed or monitored, the initiatives for urban health and wellbeing found in the document review and case studies point to a range of practices underway, and to insights that may be more widely transferable. These relate to their processes, their design, and to features that lie beyond the initiatives themselves.

#### Processes for equity-oriented change in urban wellbeing

Processes that explicitly integrate measures and tools for participatory equity and recognitional equity are pivotal, as they appear to be entry points for gains in other dimensions of equity. The power imbalances in current urban contexts call for rights-based and social accountability approaches that link conditions to rights and duties; for iterative stages that deepen and widen trust across actors; and for investment in social organisation, capacities and power. The measures for this include listening to and consulting affected communities from the onset and in their own settings, and exposing their lived experience. The latter can be done through various forms of mapping, including participatory assessments, where affected groups are themselves capacitated to gather, analyse and identify evidence and priorities.

Promoting participatory equity implies inclusion in design on investment in the skills and capacities of key social groups, and use of processes that explicitly strengthen community networks, collective organisation, ‘active citizenship’ and community leadership, with particular attention to often excluded and marginalised groups, such as women, young people, people with disabilities, informal workers and residents of informal settlements. Whether in informal or formal associations, or membership-driven social networks, or in service, sector or local authority committees, having elected and mandated community representatives, clear, agreed procedures and active feedback and health literacy outreach to local residents can avoid communities being silenced by procedures, and avoid representatives becoming delinked from their communities.

Monitoring, documenting and reporting the distributional changes from these measures for participatory and recognitional equity, including changes in social power and confidence, are important for strategic review of processes, and to share learning, including across different cities and countries. This not yet well integrated in initiatives. It is important for facilitating recognition of the key role of investment in these dimensions of equity for leveraging other distributional and structural equity outcomes.

#### Designing initiatives for equity-oriented change in urban wellbeing

The findings indicate that initiatives for urban wellbeing operate in complex contexts, often confronting longstanding deficits and inequalities exacerbated by recent trends, including climate, pandemic and other shocks. Such complex problems are not solved in siloes. Improving equity calls for measures that stimulate cross sectoral, multi-stakeholder and holistic responses to these multi-dimensional drivers of inequality and deprivation, in sustained approaches and integrating strategic review. This implies pivoting from a focus on a single problem to acting on the multiple determinants of that problem, to bring together the different interventions and actors who play a role in pathways for change on the problem, and to link social, economic and ecosystem benefits from action. The findings suggest that area-based approaches offer potential for such co-design and co-located approaches, but still merit wider further application in urban practice.

In more issue, system and social group focused work, various features enable holistic approaches. They include having credible ‘brokers’ that link and leverage the contributions of the different types of actors, skills and resources and local assets; explicitly integrating processes that stimulate and build relationships, trust, partnership and collaboration in the design of initiatives; including joint training activities; and involving research, development, testing and demonstration and introduction of appropriate technologies.

Ensuring an equity lens means integrating the measures for participatory and recognitional equity previously noted, but also linking activities that bring social benefit to economic opportunity for low income communities, including through purchasing and community contracting models that formalise community roles; and embedding capacity building, skills transfer and economic opportunities for local community members, especially women and young people.

Here too there is need for more widespread regular monitoring and strategic review to assess performance and outcomes from such approaches. This can help build community, implementer and funder confidence and enable processes to respond to emerging opportunities and challenges. It can also help to build more systematic strategic assessment and shared learning on the contribution of such approaches to structural and intergenerational dimensions of equity.

#### Enabling conditions beyond individual initiatives

The urban initiatives for health and wellbeing described in this paper suggest their potential contribution to different dimensions of equity. They also highlight, however, the limitations of local level action and authorities in producing change in policies, laws and other structural dimensions of equity. The deeper policy issues affecting structural equity cannot all be addressed at the local level, especially when policies are set by central governments and when wider economic trends, including global influences, affect and generate inequality. This calls for measures beyond the local level.

For example, a number of the initiatives nurture new forms of practice and generate, test and apply new, locally relevant and affordable technologies and methods that improve social, ecological and economic wellbeing. Technology and other innovations often came from research and development by local universities, private sectors, technical, non-profit and public institutions, social enterprises and civil society, linking with communities to ensure the relevance and demonstration of innovations. However, this calls for funding of innovation, research and development and local demonstration and accessible and affordable internet and other infrastructures. While one initiative in Bwaise Kampala reported support from a domestic innovation fund, there was limited report of this, and more such funds seem to be needed.

Development aid and external project financing often provided a catalytic contribution to processes, but was also unpredictable and target-and time-bound. The sustainability and scale-up needed to have both wider and deeper impact, including on policy change, appears to depend more on local authority capacities and services and infrastructure, such as for pro-poor primary level health care, and for urban waste management, agricultural extension and other public services. Yet these services are often underfunded. While various forms of collective savings funds, seed and innovative funding measures and ‘matchmaking’ of private funders with specific groups were used to locally resource initiatives, these efforts cannot substitute adequate domestic financing of local public services and investment in the necessary local public infrastructure and local authority capacities for initiatives to flourish.

Documenting and communicating the changes achieved by initiatives, including to higher level policy and political actors, can help to build connections and alliances and to leverage wider attention, recognition and support. It can also enable exchange across practitioners, social and professional networks, within and across countries.

The paper provides evidence of a significant volume of inspiring and innovative local intervention for urban health and wellbeing, with many features supporting key dimensions of equity. Limitations found in better monitoring and documenting their distributional outcomes and pivoting from single issues to holistic and area-based approaches can be addressed at local level.

Addressing underlying drivers that emerge from the wider political economy and dimensions of structural equity that are controlled beyond local levels calls, however, for changes in national and international level policy, institutional practice and funding systems. If, as indicated in this paper, participatory and recognitional equity are key levers of equity-oriented change, then these dimensions should not be diluted as processes move from local to national and international levels, to profile local claims and experience, and to bring greater voice in such policy dialogue from local actors driving equity-oriented urban change.

## Author contributions

RL prepared the analytic framework used in the work with external review input, prepared the synthesis of the desk review findings, carried out the thematic analysis, synthesised the findings and prepared the draft full manuscript, discussion and conclusions for review and input from GM, FG, SC, CW, and FG, co-ordinated and edited the final manuscript that all authors reviewed and signed off on, and prepared revisions to reviewer comments and co-authors reviewed the revisions. RL and GM carried out searches for the document review and identified the four cities and outline for the case studies with external review. SC, DG, CW, and FG implemented and wrote the case studies with review and edit input from RL and GM. All authors agreed to be accountable for the content of the work.

## Funding

The background work in this paper was funded by International Society for Urban Health and by EQUINET funding from Open Society Policy Centre. No funds were provided for open access publication fees.

## Conflict of interest

The authors declare that the research was conducted in the absence of any commercial or financial relationships that could be construed as a potential conflict of interest.

## Publisher’s note

All claims expressed in this article are solely those of the authors and do not necessarily represent those of their affiliated organizations, or those of the publisher, the editors and the reviewers. Any product that may be evaluated in this article, or claim that may be made by its manufacturer, is not guaranteed or endorsed by the publisher.

## Abbreviations

CFHD: Civic Forum on Human Development
EQUINET: Regional Network for Equity in Health in East and Southern Africa
ESA: East and Southern Africa
KDI: Kounkuey Design Initiative
HEART: Health Equity Assessment and Response Tool
ISUH: International Society for Urban Health
PCH: Primary health care
SDI: Slum Dwellers International
UA: Urban Agriculture

## References

[1] World Health Organisation (WHO), UN Habitat. Hidden cities: Unmasking and overcoming health inequities in urban settings. Geneva: WHO Geneva and UN Habitat (2010).

[2] Loewenson R Masotya M. Pathways to urban health equity: Report of multi-method research in east and southern Africa, EQUINET discussion paper 117. Harare: EQUINET (2018).

[3] Jenkins MW Cumming O Cairncross S. Pit latrine emptying behavior and demand for sanitation services in Dar Es Salaam, Tanzania. Int J Environ Res Public Health. (2015) 12:2588–611. doi: 10.3390/ijerph120302588, PMID: 25734790 PMC4377920

[4] Prasad A Kano M Dagg K Mori H Senkoro H Ardakani MA. Prioritizing action on health inequities in cities: An evaluation of urban health equity assessment and response tool (urban HEART) in 15 cities from Asia and Africa. Soc Sci Med. (2015) 145:237–42. doi: 10.1016/j.socscimed.2015.09.031 26456133 PMC5357782

[5] Nyemba A Manzungu E Masango S Musasiwa S. The impact of water scarcity on environmental health in selected residential areas in Bulawayo City, Zimbabwe. Phys Chem Earth. (2010) 35:823–7. doi: 10.1016/j.pce.2010.07.028

[6] Musingafi MCC Manyanye S Ngwaru K. Public health and environmental challenges in Zimbabwe: The case of solid waste generation and disposal in the city of Masvingo. J Environ. (2014) 1:68–72.

[7] Bailis R Ezzati M Kammen DM. Mortality and greenhouse gas impacts of biomass and petroleum energy futures in Africa. Science. (2005) 308:98–103. doi: 10.1126/science.1106881, PMID: 15802601

[8] Chalya PL Mabula JB NgayomelaI H Kanumba ES Chandika AB Giiti G. Motorcycle injuries as an emerging public health problem in Mwanza City, Tanzania: A call for urgent intervention. Tanzan J Health Res. (2010) 12:214–21. doi: 10.4314/thrb.v12i4.55500 24409627

[9] Hopewell MR Graham JP. Trends in access to water supply and sanitation in 31 major sub-Saharan African cities: An analysis of DHS data from 2000 to 2012. BMC Public Health. (2014) 14:208. doi: 10.1186/1471-2458-14-208, PMID: 24576260 PMC3942065

[10] Chesire EJ Orago AS Oteba LP Echoka E. Determinants of under nutrition among school age children in a Nairobi peri-urban slum. East Afr Med J. (2008) 85:471–9. doi: 10.4314/eamj.v85i10.9671, PMID: 19537423

[11] Schram A Labonte R Sanders D. Urbanization and international trade and investment policies as determinants of noncommunicable diseases in sub-Saharan Africa. Prog Cardiovasc Dis. (2013) 56:281–301. doi: 10.1016/j.pcad.2013.09.016, PMID: 24267436 PMC7111622

[12] Loewenson R Godt S Chanda-Kapata P. Asserting public health interest in acting on commercial determinants of health in sub-Saharan Africa: Insights from a discourse analysis. BMJ Glob Health. (2022) 7:e009271. doi: 10.1136/bmjgh-2022-009271, PMID: 35817497 PMC9274517

[13] Chuma J Gilson L Molyneux C. Treatment-seeking behaviour, cost burdens and coping strategies among rural and urban households in coastal Kenya: An equity analysis. Tropical Med Int Health. (2007) 12:673–86. doi: 10.1111/j.1365-3156.2007.01825.x, PMID: 17445135

[14] Soura AB Mberu B Elungata P Lankoande B Millogo R Beguy D. Understanding inequities in child vaccination rates among the urban poor: Evidence from Nairobi and Ouagadougou health and demographic surveillance systems. J Urban Health. (2015) 92:39–54. doi: 10.1007/s11524-014-9908-1, PMID: 25316191 PMC4338131

[15] Zyaambo C Siziya S Fylkesnes K. Health status and socio-economic factors associated with health facility utilization in rural and urban areas in Zambia. BMC Health Serv Res. (2012) 12:389. doi: 10.1186/1472-6963-12-389, PMID: 23145945 PMC3536624

[16] WHO. Impact of COVID-19 on people’s livelihoods, their health and our food systems, Joint statement of the ILO, FAO, IFAD, WHO. (2020). Available at: https://www.who.int/news/item/13-10-2020-impact-of-covid-19-on-people's-livelihoods-their-health-and-our-food-systems (accessed November 30, 2022).

[17] Kameri-Mbote P Merok-Mutua A. Gendered impacts of responses to the COVID-19 pandemic in Kenya, School of law. Nairobi: University of Nairobi (2020).

[18] Loewenson R Colvin CC Szabzon F Das S Khanna R Schattan-Coelho V. Beyond command and control: A rapid review of meaningful community engaged responses to COVID-19. Glob Public Health. (2021) 16:1439–53. doi: 10.1080/17441692.2021.1900316, PMID: 33734007

[19] WHO. Closing the gap in a generation: Health equity through action on the social determinants of health. Final Report of the Commission on Social Determinants of Health. Geneva: WHO (2008). 10.1016/S0140-6736(08)61690-6 18994664

[20] WHO. Health as the pulse of the new urban agenda, United Nations conference on housing and sustainable urban development, Quito. Quito: WHO (2016).

[21] EQUINET. Regional equity watch 2012: Assessing progress towards equity in health in east and southern Africa. Harare: EQUINET (2012).

[22] See J Wilmsen BA. Multidimensional framework for assessing adaptive justice: A case study of a small island community in the Philippines. Clim Chang. (2022) 170:16. doi: 10.1007/s10584-021-03266-y

[23] Anderson E. The imperative of integration. Princeton, NJ: Princeton University Press (2013).

[24] Eysenck J. Systematic reviews: Meta-analysis and its problems. BMJ. (1994) 309:789–92. doi: 10.1136/bmj.309.6957.789, PMID: 7950571 PMC2541015

[25] AbuDagga A. Trouble with systematic reviews and meta-analyses. Public Citizen Health Lett. (2017) 1–4.

[26] Lee YH. Strengths and limitations of meta-analysis. Korean J Med. (2019) 94:391–5. doi: 10.3904/kjm.2019.94.5.391

[27] Loewenson R Mhlanga G Gotto D Chayikosa S Goma F Walyaro C. Learning from initiatives on equitable urban health and wellbeing in east and southern Africa, EQUINET discussion paper 127. Harare: EQUINET (2022).

[28] Chayikosa S Mware T Mumhure G Ndlovu T Mhlanga G. Initiatives on equitable urban health and wellbeing in east and southern Africa: Harare case study report. Harare TARSC. (2022). Available at: https://www.tarsc.org/publications/documents/ACE%20EQ%20Harare%20case%20studies.pdf (accessed September 30, 2022).

[29] Goma F Mhlanga G. Initiatives on equitable urban health and wellbeing in east and southern Africa: Lusaka case study report. Lusaka: TARSC (2022).

[30] Gotto D Mhlanga G. Initiatives on equitable urban health and wellbeing in east and southern Africa: Kampala case study reports. Kampala: TARSC (2022).

[31] Walyaro C Mhlanga G. Initiatives on equitable urban health and wellbeing in east and southern Africa: Nairobi case study report. Nairobi: TARSC (2022).

[32] Walnycki A Earle L Monteith W. Towards more inclusive urban health systems for refugee wellbeing: Lessons from Kampala, Uganda. London: International Institute for Environment and Development (2019).

[33] Ahmed S Simiyu E Githiri G Sverdlick A Mbaka S. Cooking up a storm: Community-led mapping and advocacy with food vendors in Nairobi’s informal settlements. London: International Institute for Environment and Development (2015).

[34] Loewenson R Mbwili-Muleya C Zulu-Lishandu I. Case study: Lusaka district health office, Zambia. Learning from international experience on approaches to community power, participation and decision-making in health. TARSC, Shaping Health. (2017). Available at: www.shapinghealth.org (accessed September 30, 2022).

[35] Mbwili-Muleya C Lungu M Kabuba I Zulu Lishandu I Loewenson R. Consolidating processes for community – Health Centre partnership and accountability in Zambia. Harare: EQUINET (2008).

[36] Monitoria Participativa Maputo (MOPA). How an app generates data that help clean-up Maputo, Making All Voices Count, Maputo. MOPA. (2017). Available at: https://www.makingallvoicescount.org/news/mopa-how-an-app-generates-data-that-help-clean-up-maputo/ (accessed August 30, 2022).

[37] World Resource Institute, Ross Centre. Kenya Kounkuey design initiative (KDI), ‘Kibera public space project. WRI. (2021) Available at: https://prizeforcities.org/project/kibera-public-space-project (accessed September 30, 2022).

[38] Kounkuey Design Initiative (2022). Available at: https://www.kounkuey.org/ (accessed August 30, 2022)

[39] Cities Alliance. Waste management for flood control in Bwaise, an urban slum in Uganda, project overview document 20202021. Cities Alliance (2022). Available at: https://www.citiesalliance.org/sites/default/files/2021-08/FinalReport_CfP20_TAU%20Uganda.pdf (accessed August 30, 2022).

[40] HEPs Uganda. Becoming visible: Tackling the urban health divide in Kawempe slum, Kampala, Uganda. Kampala: HEPS (2012).

[41] Training and Research Support Centre (TARSC) and Civic Forum on Housing. Assessment of solid waste management in three local authority areas of Zimbabwe, report of a community-based assessment. Harare: TARSC (2010).

[42] Food and Agriculture Organisation (FAO). City-to-city cooperation for sustainable urban food systems. New York, FAO: (2021).

[43] Halliday J Platenkamp L Nicolarea Y. A menu of actions to shape urban food environments for improved nutrition. GAIN, MUFPP and RUAF. (2019) Available at: https://foodactioncities.org/app/uploads/2021/06/LCS1_Quelimane_Improving_Food_Access.pdf (accessed September 30, 2022).

[44] Equity Gauge Zambia and Lusaka District Health Board. Strengthening community – Health Centre partnership and accountability in Zambia. Harare: EQUINET (2006).

[45] Kitale L Zella AY. Greenhouse farming of vegetables and its contribution to urban food security and farmers’ wellbeing: A case study of Dar Es Salaam, Tanzania. J Agric. (2022) 9:1–10.

[46] Banana E Chitekwekwe-Biti B Walnycki A. Co-producing inclusive city-wide sanitation strategies: Lessons from Chinhoyi. Zimbabwe Environ Urban. (2015) 27:35–54. doi: 10.1177/0956247815569683

[47] Broto VC Salazar D Adams K. Communities and urban energy landscapes in Maputo, Mozambique. People Place Policy. (2014) 8:192–207. doi: 10.3351/ppp.0008.0003.0005

[48] Broto CV. Delivering sustainable energy in urban Mozambique. London: British Academy (2019).

[49] Oates L Gillard R Kasaija P Sudmant A Gouldson A. Supporting decent livelihoods through sustainable service provision: Lessons on solid waste management from Kampala, Uganda. London: Coalition for Urban Transitions (2019).

[50] Few R Harpham T Atkinson S. Urban primary health care in Africa: A comparative analysis of city-wide public sector projects in Lusaka and Dar Es Salaam. Health Place. (2003) 9:45–53. doi: 10.1016/S1353-8292(02)00029-1, PMID: 12609472

[51] Kounkuey Design Initiative (KDI). Architectural Association of Kenya, Institute for Transportation and Development Policy. Technical brief on Kibera Road developments KDI. (2021). Available at: https://www.kounkuey.org/uploads/1700017/1618917898194/Technical_Brief_on_Kibera_Road_Developments_2021_AAK-KDI-ITDP_1_1_2.pdf (accessed September 30, 2022).

[52] Stewart R Korth M Langer L Rafferty S Da Silva NR van Rooyen C. What are the impacts of urban agriculture programs on food security in low and middle-income countries? Environ Evid. (2013) 2:7. doi: 10.1186/2047-2382-2-7

[53] Hivos. The Zambian food change lab: Jointly identifying solutions to Zambia’s food systems challenges. Lusaka: Hivos (2020).

[54] Barden-O'Fallon J Evans S Thakwalakwa C Alfonso W Al J. Evaluation of mainstreaming youth-friendly health in private clinics in Malawi. BMC Health Serv Res. (2020) 20:79. doi: 10.1186/s12913-020-4937-9, PMID: 32013943 PMC6998314

[55] Makadzange K Radebe Z Maseko Lukhele V Masuku S Fakudze G Mengestu TK. Implementation of urban health equity assessment and response tool: A case of Matsapha, Swaziland. J Urban Health. (2018) 95:672–81. doi: 10.1007/s11524-018-0241-y, PMID: 29616450 PMC6181813

[56] Croese S Dominique M Raimundo IM. Co-producing urban knowledge in Angola and Mozambique: Towards meeting SDG 11. Urban Sustain. (2021) 1:8. doi: 10.1038/s42949-020-00006-6

[57] Muchadenyika D. Slum upgrading and inclusive municipal governance in Harare, Zimbabwe: New perspectives for the urban poor. Habitat Int. (2015) 48:1. doi: 10.1016/j.habitatint.2015.03.003

[58] Loewenson R Laurell AC Hogstedt C D’Ambruoso L Shroff Z. Participatory action research in health systems: A methods reader, TARSC, AHPSR, WHO, IDRC Canada. Harare: EQUINET (2014).

[59] Giugliani C Duncan BB Harzheim E Lavor ACH Lavor MC Machado MMT. Community health workers programme in Luanda, Angola: An evaluation of the implementation process. Hum Resour Health. (2014) 12:68. doi: 10.1186/1478-4491-12-68, PMID: 25491732 PMC4292814

[60] Andrianantoandro VT Pourette D Rakotomalala O Ramaroson HJV Ratovoson R Rakotoarimanana FMJ. Factors influencing maternal healthcare seeking in a highland region of Madagascar: A mixed methods analysis. BMC Pregnancy Childbirth. (2021) 21:428. doi: 10.1186/s12884-021-03930-2, PMID: 34134653 PMC8210351

[61] Oxfam. Citizen voice in Zambia evaluation of the ‘vote health for all’ campaign. Oxford, UK: Oxfam GB (2013).

[62] Monitor. What it means to work in flooded Bwaise. Monitor Uganda edition January 5, (2021). Available at: https://www.monitor.co.ug/uganda/lifestyle/reviews-profiles/what-it-means-to-work-in-flooded-bwaise-1511100 (accessed September 30, 2022).

[63] Lwasa S. Uganda offers lessons in tapping the power of solid waste. The conversation. (2019). Available at: https://theconversation.com/uganda-offers-lessons-in-tapping-the-power-of-solid-waste-119728 (accessed September 30, 2022).

[64] Lusaka District Health Office, TARSC. Using photovoice to strengthen health centre committees as a vehicle for social participation in health system in east and southern Africa: Zambia report. Harare: EQUINET (2016).

[65] Cravero P. Informal food vendors: Urban food security's invisible experts. London: International Institute for Environment and Development (2015).

[66] Cloete C Hampton J Chetty T Ngomane T Spooner E Zako LMG. Evaluation of a health system intervention to improve virological management in an antiretroviral programme at a municipal clinic in Central Durban. Southern African J HIV Med. (2019) 20:985. doi: 10.4102/sajhivmed.v20i1.985, PMID: 31616575 PMC6779997

[67] Kasinja C Tilley E. Formalization of informal waste pickers’ cooperatives in Blantyre, Malawi: A feasibility assessment. Sustainability. (2018) 10:1149. doi: 10.3390/su10041149

[68] Kanonhuwa TN Chirisa I. Food waste in urban Zimbabwe: Options for food recycling In: Toriro P Chirisa I, editors. Environmental resilience. Advances in 21st century human settlements. Singapore: Springer (2021)

